# Potency-Enhancing Synthetics in the Drug Overdose Epidemic: Xylazine (“Tranq”), Fentanyl, Methamphetamine, and the Displacement of Heroin in Philadelphia and Tijuana

**DOI:** 10.31389/jied.122

**Published:** 2022-12-02

**Authors:** FERNANDO MONTERO, PHILIPPE BOURGOIS, JOSEPH FRIEDMAN

**Affiliations:** Columbia University, US; University of California, Los Angeles, US; University of California, Los Angeles, US

**Keywords:** Xylazine, fentanyl, methamphetamine, polysubstance use, narcotics supply chains, synthetic drugs, tranq

## Abstract

Multiple transformations—referred to as “waves” in a panoply of recent public health and law enforcement publications—have rendered North American drug markets increasingly toxic since the early 2010s. The introduction of exceptionally potent synthetic sedatives and stimulants is initiating a new generation of drug injectors into co-use of opioids and methamphetamine, catapulting rates of deadly overdoses and infectious diseases. Drawing on extensive participant-observation research in Philadelphia (2007-present) and Tijuana (2018-present), we document the experience of street-based drug users across these two North American cities to focus on regional shifts in narcotics supplies and endpoint user preferences. We link the dramatic proliferation of fentanyl, methamphetamine, xylazine, and Mexican white powder heroin to: 1) pre-existing drug supply networks on the western and eastern coasts of the North American subcontinent; 2) material characteristics of local heroin supplies in pre-fentanyl opiate markets (Mexican black tar vs. Colombian off-white powder heroin); and 3) racialized repression/incarceration of drug sellers and users on both sides of the Mexico-US border. The article combines economic and medical anthropology to develop an ethnographically-informed political economy approach to an urgent public health challenge among street-based drug users with the highest overdose mortality rates in the US Northeastern Rust Belt and the Northwestern Mexican borderland metroplex anchored by Tijuana. It foregrounds street users’ experiences in real time amidst rapidly shifting narcotics supply chains, linking market-driven logics of profit-seeking to the war on drugs’ prohibitionist policy context, highlighting increasing toxic impacts on vulnerable sectors across regions.

Multiple transformations—referred to as ‘waves’ in a panoply of recent public health and law enforcement publications (Ciccarone 2021; McCann Pineo and Schwartz 2020; DEA 2021: 2)—have rendered narcotics markets in Mexico, the US, and Canada increasingly toxic since the early 2010s. The introduction of exceptionally potent synthetic sedatives (fentanyl and xylazine) and stimulants (particularly ‘crystal’ methamphetamine) is initiating a new generation of drug injectors into new varieties of polysubstance use, catapulting rates of deadly overdoses, infectious diseases, and interpersonal violence. Unfortunately, critical public health and social science analyses have had only limited impact linking systemic knowledge of the socioeconomics of narcotics markets to the political and public health implications of these changes, for two particular reasons.

Firstly, decades-old paradigms and practices of narcotics prohibitionism circumscribe the evidential basis and analytical scope of scholars and service practitioners in both public health and the social sciences. Law enforcement agencies produce flawed, belated surveillance reports on drug markets while restricting access to their raw data and criminalizing the efforts of independent researchers to collect information via participant-observation methods. Street-level police officers habitually harass social science ethnographers and public health outreach workers near open-air narcotics markets, detaining them for questioning and sometimes brutalizing and arresting them (Friedman et al. 2019). Law enforcement agents routinely repress the distribution of life-saving harm reduction technologies such as fentanyl test strips and naloxone (Narcan), deeming them illegal ‘drug paraphernalia’ (Carroll et al. 2022). District attorneys (prosecutors) throughout the US rely on the plea-bargaining system to increase the efficiency and lower the cost of handling their extensive caseloads (Gramlich 2019). This has the effect of circumventing trials and thus minimizing third-party evaluation of prosecutorial evidence. By pressuring defendants to accept plea bargains in exchange for shorter sentences, prosecutors avoid subjecting narcotics charges to the scrutiny of juries and judges. In this manner, they have curtailed the need for detailed knowledge about drugs and even basic familiarity with narcotics logistics in the criminal justice system.

Second, researchers become understandably wary of sensationalist media and are reluctant to document the socioeconomic organization of narcotics markets. Some scholars mistakenly assume that detailed knowledge about drug economies plays a key role in policing and in the criminal justice system, and warn drug researchers that their findings may inadvertently contribute to punitive interventions. Internal review boards evaluating qualitative, non-clinical research on ‘human subjects’ regularly subject studies of drug markets to non-sequitur ‘ethical’ logjams, further deterring participant-observation research on criminalized practices. Longstanding intellectual traditions linked to humanism, liberalism, and even Marxism continue to steer scholars away from the study of criminalized practices as if they were undignified or ethically suspect.

A long-term effect of these dynamics is that mainstream public health and social sciences research on illegal drug use in the United States has narrowed its analytical scope to the realm of individual drug use behaviors. More specifically, the prevailing line of analysis in these fields revolves around micro-behaviors to be altered through the provision of services, information and silver-bullet technologies to individual users. Meanwhile, key questions of political and public health import concerning drug *selling*, mixing, adulteration, and transportation are relinquished to criminal justice agencies, especially the Drug Enforcement Administration (DEA) and local police forces. In other words, public health has been relegated to downstream management of individualized pathologies, while law enforcement agencies monopolize the upstream assessment of narcotics supply chains.

Yielding this upstream terrain to law enforcement agencies has proven disastrous for drug users and sellers alike. Despite a historically unprecedented explosion of powerful synthetic substances across North America in the 2010s and 2020s, the DEA has repeatedly bungled testing and supply monitoring and delayed or forbidden dissemination of its data. Due to its longstanding, racialized hypercriminalization of the petty dealer, the DEA exaggerates the responsibility of retail sellers for toxic adulteration of the product they sell (e.g., DEA 2020: 10; DEA PFD 2020a: 7–8). The important distinctions between global, regional, local, and neighborhood-level narcotics markets remain practically unexamined. Lacking crucial supply-level data, public health is unable to respond in real time to critical transformations in narcotics supply chains. Instead, street sellers are mistakenly blamed for “poisoning” their customers with adulterants such as fentanyl, a displacement of responsibility that has revived the punitive thrust of US drug policy at a moment when the tide appeared to be shifting in favor of public health logics.

Public health, the humanities, and the social sciences have not pushed back against these developments with sufficient vigor or imagination. For example, at what level of the supply chain does fentanyl and/or xylazine enhancement actually take place? Public health should already have robust answers to this question instead of having passively ceded this terrain to law enforcement’s punitive logics. Anthropologists and other scholars with expertise in participant-observation methods and political economy analysis could significantly expand the reach of harm reduction by more systematically analyzing drug sales and supply chains in addition to users’ vulnerabilities and pharmacological preferences. Drug retailers represent an untapped potential conduit for population-level, structurally and culturally competent, micro-community-based, supply-level harm reduction priorities. The current division of labor in the production of knowledge about drugs, however, has forestalled unconventional interventions that would incorporate systemic knowledge of the socioeconomics of drug markets.

This article brings together the study of macro and micro drug supply chains and the analysis of endpoint drug consumption by combining participant-observation ethnography and a close reading of the often inaccurate, always outdated, publicly available DEA surveillance data. We combine the tools of medical and economic anthropology to examine the recent shifts in the drug markets of two sentinel cities: Philadelphia and Tijuana, where we have long-term ethnographic and epidemiological collaborations.

Most of the recent changes in North American narcotics markets involve the displacement of agriculturally produced substances such as poppy-sourced heroin and coca-bush sourced cocaine by the synthetic sedatives and stimulants fentanyl, xylazine, and methamphetamine. Drawing on team-based, participant-observation research among street-level drug sellers and injectors in both Philadelphia (2007–present) and Tijuana (2018–present), we reinterpret US local, state, and federal forensic drug monitoring reports from 2013–2022 to analyze how synthetic sedatives and stimulants, in combination with the fleeting supremacy of Mexican white powder heroin from 2014–2019, have transformed narcotics markets across the subcontinent—especially the US Northeastern Rust Belt and the Northwestern Mexican borderland metroplex anchored by Tijuana.

Our findings link the dramatic proliferation of fentanyl, methamphetamine, xylazine, and Mexican white powder heroin to: 1) pre-existing drug supply networks on the western and eastern coasts of the North American subcontinent; 2) material characteristics and consumption logistics of local heroin supplies in pre-fentanyl opiate markets (Mexican black tar vs. Colombian off-white powder heroin); and 3) racialized repression, deportation, and incarceration of drug sellers and users on both sides of the Mexico-US border. We offer what we believe to be the first data-driven rebuttal to the DEA’s thesis that fentanyl adulteration takes place predominantly at the level of street retail. In sentinel distribution cities with open-air drug markets like Philadelphia, fentanyl enters the street supply at the level of local wholesale—two steps upstream from the level of street retail sales.

## THE CHANGING LANDSCAPE OF NORTH AMERICAN DRUG MARKETS: THE TOXIC EFFECTS OF PROHIBITIONISM AND DRUG CAPITALISM

The first major transformation in the last decade began in 2012/2013, when the synthetic opioid fentanyl entered heroin supply chains as a potency-enhancing adulterant (Ciccarone 2019; Armenian et al. 2018). Fentanyl and its analogues (henceforth ‘fentanyl’ for brevity) can be 30 to 10,000 times more potent than heroin. Already historically high, overdose mortality rates rose to historically unprecedented levels in the United States and its two neighbors on the North American subcontinent, Mexico, and Canada.

A second, simultaneous transformation received surprisingly little attention outside of relatively obscure and often inconsistent US Drug Enforcement Administration’s (DEA) annual reports monitoring wholesale and retail drug markets. Having reached the levels of quality and solubility to which heroin users had become accustomed in the eastern half of the United States, Mexican powder heroin supplanted Colombian heroin as the principal poppy-sourced opiate sold in that region between 2014 and approximately 2019 (DEA 2020b; DEA 2019; DEA PFD 2020a).

A third change, occurring unevenly throughout the 2010s but accelerating in 2015, has involved the diffusion of highly potent, low-cost Mexican crystal methamphetamine across North America (Han et. al 2021b). This powerful stimulant supplanted domestic methamphetamine and appears to be out-competing crack and cocaine in regional markets across the United States.

Finally, starting in the late 2010s, several US cities with large, segregated Puerto Rican neighborhoods such as Philadelphia and parts of Connecticut witnessed the appearance of a new opioid enhancer, the veterinary tranquilizer xylazine (Friedman, Montero & Bourgois 2022). Known as ‘tranq’ among street-based users, xylazine is an exceptionally potent sedative/tranquilizer designed for heavy animals (Ruiz-Colon et al. 2014). As a heroin adulterant, it was first documented in rural Puerto Rican drug markets located near cattle-ranching and thoroughbred race-horse breeding towns (Rodriguez et al. 2008; Reyes et al. 2012; Torruella 2011), but it is spreading across the US Rustbelt mainland. In cities like Philadelphia, the advent of xylazine has helped to complete the definitive displacement of heroin from the local opioid supply. Recent spectrometer drug testing data compiled by the Center for Forensic Science Research and Education (CFSRE) in partnership with the Philadelphia Department of Health show that the average ‘dope’ sample from Philadelphia today consists of approximately 30–40% xylazine and about 2–10% fentanyl (Whelan 2022; CFSRE 2022a; CFSRE 2022b). Out of 187 ‘dope’ samples tested by CSFRE between March and September of 2022, only 6 contained significant amounts of heroin. In other words, xylazine increasingly comprises the bulk of the substance that street-based sedative users consume in Philadelphia.

These shifts are having devastating economic and health impacts on the most vulnerable actors across the North American narcotics production and supply chain—notably, small farmer producers, unemployed polysubstance-using consumers, and racialized street retail sellers. They have been churned into profitably expendable fodder as a result of the ‘war on drugs.’ Critical medical anthropologists, geographers, and public health scholars have shown that prohibitionist interventions such as crop eradication programs in Mexico and South America fomented the shift by wholesale drug producers to fentanyl and methamphetamine production labs, paralleling the global industrialized for-profit logics of the legal economy (Karandinos 2018; Karandinos 2017; Beletsky & Davis 2017; Le Cour Grandmaison, Morris & Smith 2019). Synthetic chemical labs require miniscule labor forces and infrastructural footprints compared to the sprawling territories necessary for growing and processing poppy plants and coca bushes and the bulky techniques of extracting psychoactive components from agricultural raw materials in remote territories. Synthetic narcotics are less bulky, facilitating smuggling and processing. They are also less labor-intensive and are impervious to the droughts, pests, and soil fertility declines that characterize agricultural production in the era of climate change.

A key underexamined feature of the US war on drugs is the renunciation by state institutions to any active role in the quality control of public consumables. Heroin’s potency-enhancing adulteration with xylazine and synthetic fentanyl analogues serve as a vivid illustration of how drug prohibitionism has fomented a toxic regulatory regime negatively impacting laborers and consumers while enhancing capital accumulation. In the United States, Latin and African American street dealers (labor) are subject to incessant punitive policing and mass incarceration, while the investors and purveyors of wholesale narcotics at the local, regional, and international levels, including the financial institutions laundering narcotics profits (i.e., capital) are left relatively untouched. Even a superficial skim of the US government’s official reports confirm that the narcotics seized by US law enforcement agencies are a mere drop in the bucket compared to the exorbitant quantities of narcotics reaching North American endpoint consumers annually. Most ironically, a complex machinery of financial regulation allows the profits from illegal narcotics sales to enter the banking system at relatively minimal economic cost or legal risk (Farfan-Mendez 2019; Woodman 2020; Tatone 2020).

Adulteration and potency enhancement are only one set of pharmacological marketing and production practices characterizing the ‘fourth wave’ of synthetic sedatives and stimulants. In addition to adulteration, this article will elaborate on other key practices—including diversification, substitution, counterfeiting, refinement, and supplementation—giving structural shape and political economic logic to US drug markets and shifting street-user preferences during the late 2010s and early 2020s. We link descriptions of each relevant substance to users’ accounts of their experience with those substances and discuss their public health impact. The article is divided into four sections, one for each substance (powder heroin, methamphetamine, fentanyl, and xylazine). The first three sections contain two subsections, one for each of the two cities where we have conducted the most extensive, long-term participant observation.

## MEXICAN POWDER HEROIN

By far the least reported transformation in North American drug markets over the last decade was the displacement of Colombian powder heroin by Mexican powder heroin east of the Mississippi River between 2014 and approximately 2019. This change is also the most poorly understood. Inconsistent DEA data reports and faulty DEA testing and categorization methods since 2014 delayed awareness of the implications of this shift for both the US and Mexico by almost a decade. As early as 2011, the DEA’s annual Heroin Domestic Monitoring Program report noted in passing that Mexican powder heroin was ‘expanding… into markets traditionally dominated by SA heroin [South American, i.e., Colombian]… in the eastern and midwestern US’ (DEA 2013: 11). It was not until 2014, however, that DEA developed the technical capacity to detect Mexican powder heroin produced by emulating Colombian methods, a new heroin source-type that some public health scholars refer to as a ‘Mexican mimic’ of Colombian heroin (Ciccarone 2019: 186). That same year, the DEA finally introduced the signature ‘MEX-SA’ in its annual reports to classify Mexican heroin produced using methods formerly employed only in Colombia.

This new heroin source-type was a significant technological innovation for Mexican producers, who formerly monopolized US markets west of the Mississippi with a visually distinct ‘black tar’ heroin formulation (gooey or waxy depending on temperature). Black tar continues to dominate most opioid markets in the western half of the United States, whereas Mexican powder heroin briefly overtook the eastern US until 2019 (assuming the DEA’s testing was accurate during those ‘MEX-SA’ dominated years).

It is difficult to determine the exact moment when this change took place, but DEA reports claim Mexican powder heroin supplanted Colombian powder heroin east of the Mississippi by 2014. According to the DEA’s 2014 ‘Heroin Signature Program’ (HSP) report, based on data drawn from wholesale seizures (surpassing 1 kg) at the border and elsewhere throughout the country, Mexican powder heroin accounted for 58% (by weight) of the heroin analyzed by the program that year, whereas Colombian heroin had dropped to only 17% (DEA 2016: 5). In contrast, in 2009, Colombian heroin had represented 62% of the heroin seizures analyzed by that same ‘Heroin Signature Program’ (DEA 2016: 13). By 2018, Mexican-origin heroin in both its powder and black tar formulations accounted for 93% of heroin seizures, while Colombian heroin plummeted to 2% (DEA 2020: 3).

Scholars, journalists, and government officials initially assumed that Mexican powder heroin was closely associated with fentanyl at the international wholesale level. DEA data on opioid confiscations from 2013–2020, however, show that Mexican powder heroin and fentanyl enter the United States along two distinct supply chains. In 2016, for instance, out of 395 large seizures of powder heroin analyzed by DEA’s HSP program, less than 2% contained fentanyl. None of the powder samples seized at Southwest border ports of entry contained any fentanyl (DEA 2020: 10). (Once again, this assumes comprehensively accurate DEA testing logistics.) Despite the fact that the number of fentanyl-adulterated heroin seizures grew every subsequent year until 2018, the last year when national-level data have been made publicly available, only 23% of the powder heroin seized in wholesale volumes in 2018 contained ‘some’ fentanyl. The DEA tested 372 heroin samples in 2018 and only fentanyl-laced samples came from Southwest border entry-points (DEA 2020: 10). These data suggest that Mexican-based drug smugglers were not systematically mixing fentanyl with powder heroin inside Mexico at wholesale levels.

Our separate supply chain thesis is unambiguously substantiated at regional wholesale levels in the US Northeast. The DEA’s Philadelphia Field Division’s 2020 report detected no fentanyl in ‘all heroin seizures greater than five kilograms in Pennsylvania between 2017 and 2019’ (DEA PFD 2020a: 5). Likewise, no heroin was found in ‘100 percent of fentanyl seizures greater than five kilograms’ (DEA PFD 2020a: 7). In other words, data on heroin and fentanyl wholesale confiscations reveal that, by and large, each substance travels separately until reaching local, subregional wholesale supply chains.

While DEA national and state-level reports repeatedly affirm that heroin’s adulteration with fentanyl occurs at the micro-retail street level (see, for instance, DEA 2020: 10; DEA PFD 2020a: 7–8), this is an incorrect assumption shaped by the US war on drugs’ racialized hypercriminalization of petty street dealers. Our team’s 14 years of direct ethnographic observation in Philadelphia included thoroughly triangulated conversations with multiple drug corner owners, managers, and sellers. These conversations consistently revealed that retailers in Philadelphia do not know their product’s contents, because they buy their opioid and stimulant supplies from local wholesale suppliers already prepackaged in the smallest denomination packets. In fact, their ignorance about the content of their product makes them anxious about competitors with potentially superior product. Clearly, fentanyl adulteration in Philadelphia is not occurring at the micro-retail street level, but at the local wholesale neighborhood level. This level primarily operates in suburban houses owned and controlled by third parties where substances are mixed and packaged for the downstream retail market. State intervention, punitive or otherwise, is practically inexistent at this level of the drug supply. Ethnographic findings in Massachusetts (Ciccarone, Ondocsin & Mars 2017: 149) and Huntington, West Virginia (unpublished data) suggest that this conclusion is true for those locations as well.

Why did Mexican powder heroin supplant Colombian heroin? At the time of Colombian heroin’s definitive displacement from the US mainland in 2014–2015, Mexican powder heroin was already purer and cheaper than its Colombian counterpart at both the wholesale and retail levels (DEA 2016: 6, 8–9; DEA 2017: 2). Regional wholesale purveyors might have shifted by linear economic logic to this purer and cheaper Mexican product. Furthermore, Mexican heroin’s rise to dominance coincided with the relative collapse of Caribbean smuggling routes in the early 2010s, noted in DEA and UN reports (e.g., DEA 2021: 35) and confirmed by the first author’s (FM) ethnographic observations on wholesale drug commerce in the Caribbean region (Montero Forthcoming). In contrast to South American cocaine, Colombian heroin has historically been smuggled exclusively through the Caribbean archipelago in relatively small volumes (Paoli et al. 2009: 161–180), rendering heroin supply chains vulnerable to the significant weakening of Caribbean smuggling routes in the early 2010s. During these years, South American smugglers shifted northbound drug smuggling routes primarily to the Pacific Ocean, which is larger and more difficult to patrol than the Caribbean Sea. Heroin supply chains within Colombia might not have adapted to this shift quickly enough to survive the changes it prompted in North American opioid markets closer to retail endpoints. One set of authors (Mars, Rosenblum & Ciccarone 2018: 777; see also UNODC 2017) suggest that a shortage of Colombian heroin in the US in the late 2000s to early 2010s contributed to the emergence of a rival form of heroin and fomented experimentation with the addition of potency-enhancing adulterants, such as fentanyl, during those years.

Further dialogue with ethnographers in Mexico and Colombia (see articles in this special issue) can elucidate the fuller range of circumstances driving such momentous transformations of the hemispheric heroin supply. The public health crisis and economic dislocations inflicted on the most vulnerable sectors among source-producers, transshipment points, and endpoint retailers and consumers have been overwhelming. For instance, it is likely that the highly refined form of Mexican powder heroin that transformed opioid markets in the eastern half of the United States requires smaller quantities of poppy paste for its production than traditional Mexican black tar. The crisis in poppy production in the Mexican states of Michoacan and Guerrero (see Le Cour Grandmaison, Morris & Smith 2019) might have been triggered by the Mexican technological shift to powder heroin, which facilitated subsequent enhancement with fentanyl. Conversely, Mexico’s temporary domination of the US heroin market may have briefly reversed regionally uneven declines in Mexican poppy production. The growing spread of xylazine across the Eastern and midwestern half of the United States, however, suggests the potential advent of a post-agricultural heroin era, which would further undercut Mexican small-farmer poppy growers.

## MEXICAN POWDER HEROIN IN PHILADELPHIA

Users and street sellers in Philadelphia are completely unaware that this particular change occurred. From 1991 to approximately 2014, heroin in the city remained almost exclusively Colombian origin. Nevertheless, users touted their product as ‘China white’ because of its slightly off-white powder appearance, consistent with Hollywood blockbuster film portrayals of 1970s–1980s Southeast Asian heroin. This imprinted loyal ‘brand recognition’ in opioid user nomenclature and mythology (Ciccarone and Bourgois 2003). It was only as a result of fentanyl’s emergence that a public health discourse about supply changes entered the street-based user scene through Philadelphia’s harm reduction non-profit Prevention Point, which is located in the user hotspot of Kensington Avenue. This important harm reduction-informed publicized the distinction between heroin, fentanyl, and xylazine, but never noticed the Colombian to Mexican powder heroin shift.

For the first time since we started conducting research in Philadelphia, users in 2021 referred to heroin as ‘brown,’ a reference to Colombian off-white powder heroin that used to turn brown when mixed with water immediately prior to injection. The term ‘brown’ is now used interchangeably with the terms ‘heroin,’ ‘real heroin,’ and ‘dope-dope’ to differentiate ‘real’ heroin from the known adulterants fentanyl (a.k.a. ‘fent,’ ‘fentyn,’ and ‘fentanyl’) and xylazine (a.k.a. ‘tranq,’ sometimes ‘krokodil’). The term ‘dope’ has become an ambiguous catchall term referring to heroin, fentanyl, and xylazine in any of their permutations. When asked in the spring of 2021 if they knew the origin of the ‘heroin’ they were injecting, users gave wildly varying answers ranging from Puerto Rico, Dominican Republic, and Colombia to distant China.

The most surprising feature of the ‘fourth wave’ of synthetic opioids and stimulants has been the relative resilience of powder heroin in many towns and cities east of the Mississippi. It took five or six years for fentanyl to fully displace heroin from street drug markets in this part of the country. In the last DEA report listing municipal-level data (published in 2018 with data from 2016), 97% of Philadelphia’s retail samples were classified as ‘MEX-SA [Mexican powder heroin]’ with average purity of 64.7% (DEA 2018: 15). This exceptionally high level of purity for the fentanyl era surpassed the average purity of heroin in any other US city, with several regional cities like Pittsburgh (50.7% average purity), Newark (55.3%), New York (45.8%), and Atlanta (50.5%) also being relatively pure (DEA 2018: 21). In contrast, cities like Chicago (19.5% average purity), Boston (17.2%), and Baltimore (30.1%) had much lower levels of heroin purity, as one would expect in the fentanyl era (DEA 2018: 21).

The high purity levels of Mexican powder heroin in the Philadelphia opioid supply between 2014–2019 was reflected in users’ appreciation of the pharmacological effects of their ‘high’ during those years. Users reported ‘staying well,’ relaxedly free from opioid withdrawal symptoms, following habitual heroin injections for 7–8 hours, compared to 1–2 hours in other parts of the country such as the former mill towns of Massachusetts (Ciccarone, Ondocsin & Mars 2017: 149). Heroin has a much longer metabolic half-life than fentanyl, resulting in a longer high that users very much enjoy compared to fentanyl’s short-lived action. From 2014–2019, Philadelphia users’ descriptions of a lengthier buzz resulted from the relatively high concentrations of heroin in the city’s fentanyl-laced supply. Today, users report an average high lasting 5–6 hours. This higher-than-average duration appears to be linked to widespread xylazine adulteration (see below).

Why did fentanyl not immediately supplant powder heroin in the opioid supply of cities like Philadelphia, Newark, and Atlanta? On the one hand, heroin simply continues to be a prized opiate. Veteran users of heroin in Philadelphia nostalgically describe the deep, holistic, full-body embrace of ‘brown,’ which offered them 8–12 hours of comfort before the onset of withdrawal symptoms. ‘I miss real heroin’ was still a common refrain in the spring of 2021. In addition, there is a competitive urban street-corner logistics to Philadelphia’s open-air drug market that fosters high quality levels in the retail supply. Street-based people who use drugs are concentrated in public spaces such as sidewalks, subway stations, and local parks. This urban logistics facilitates a robust information network sensitive to constant variations in corner-to-corner retail offerings. When asked how they select a particular corner to buy their drug of choice, a typical user replied: ‘We usually ask around like “what’s good” and “how long ago did you do it?” Because even if they give [free] samples in the morning and it’s good stuff, when you go back to buy it later it’s not always the same quality. So you’ve gotta ask everyone who’s doing it around you, “What’s good? What’s good?” and then “How long ago?” Depending on what they say, that’s where we go.’ This information network shapes demand, which in turn has a regulatory impact on supply, moderating local levels of adulteration.

This collective regulatory impact remains limited, however, and no matter the romantic memories of ‘real’ 8–12-hour long heroin highs, fentanyl and xylazine pervade the contemporary heroin supply and are changing predilections for generational drugs of choice that also tend to be racialized. In mid-2021, users generally informed each other about which corner’s current supply most resembled heroin or ‘brown,’ versus which one was more ‘tranqy,’ versus ‘tranq-fentyn’ or ‘pure fent.’ Hence a statement by one user for an option that did not exist four years ago: ‘My favorite is tranq-fentyn with some brown.’

## MEXICAN POWDER HEROIN IN TIJUANA

The diversity of the opioid supply in Northwestern Mexico’s street markets distinguish them from US markets. While US markets are still loosely divided into a ‘black tar heroin’ West and a ‘powder dope’ East, street markets in Tijuana consistently offer both types, with white powder rising in popularity. Mexican users were initially unaware of the term fentanyl. Over the course of our fieldwork, the word ‘fentanilo’ became commonly used and often represented a term for sellers to advertise their product as having a high potency. Tijuana’s diverse supply may result from the city’s function as a major transit hub and storage zone for narcotics passing into the United States from different regions. Just as in the US, however, only powder heroin is habitually adulterated with fentanyl. On the contrary, black tar is a solid resin known as ‘goma negra’ that is logistically difficult to alter without transforming its color and consistency. While black tar has predominated for decades, white powder heroin, also known as ‘China white’ or ‘la china,’ arrived approximately in 2016. Users affirm that ‘la china’ has increased in potency and availability over the past five years. (We are yet unable to determine the changes in relative concentration of heroin and fentanyl in this local powder supply due to unavailability of data.) In the street market, this powder is usually white, although it sometimes comes in shades of brown or even bright yellow or orange depending on cutting agents, processing methods, or dyes purposefully added for brand recognition.

Healthcare providers link ‘China white’ to increased risk of overdose (OD) and soft tissue infections (abscesses). Tijuana injection drug users recognize the increased OD and other health risks, but simultaneously praise its psychoactive potency:

‘Man with goma I can do waaaaaay more, like almost 2 full [50 peso] bags. Pero la *china es muy mala, con la mitad de una 50 se puede doblar* [with half a 50 peso bag you can overdose (50 pesos = US$2)]. The first time I used “la china”, I almost died. Well it’s not real China white, but that’s what they call it—it’s like fentanyl or some shit from the East Coast…I was surprised to see that when I got deported here [2 years prior]. But don’t get me wrong, that shit is strong, cause it’s just pure fentanyl or whatever. And it’s fun, I’m not going to lie. I’m fully into it now, it’s the only shit I use, unless they run out and I have to do black for a bit. And yeah, the China rots your body from the inside out, and that’s why you see all these people out here doblando [ODing] with flesh and bone hanging out, but the rush is just too good.’

Marta, F, 45, injects opioids and methamphetamine

Strong preferences are often expressed for both varieties of opioids. Older veteran drug users who have used black tar heroin for decades often prefer its psychoactive effects. They describe it as ‘more pure’ and ‘real heroin – not that fake crap [fentanyl].’ They also often claim it represents a safer choice, less likely to cause overdose and other health risks, including soft-tissue infection:

‘Look man, I’ve saved like 12 people with bloqueadores [Narcan] this year. And I’m always the one who has to do it, cuz I’m the one smoking black. But all these other fools out here are slamming china and *doblando* like twice a week…minimum! And plus, goma is just better. It’s got this better feeling to it…it’s a big difference. Yeah it’s more expensive, but it’s safer, and I’m not tryin’ to fuck around with that fake shit.’

Eduardo, M, 45, smokes and sniffs opioids

Seeking to maximize the psychoactive effect per peso spent on opioids, younger drug users typically prefer powder formulations. They universally describe ‘China white’ as the most potent option—even those who prefer black tar’s ‘warmer’ psychoactive embodied effects acknowledge ‘la china’s’ potency. It is usually described as shorter-acting, but some insist it staves off abstinence symptoms for just as long. This may reflect the distinct array and concentration of multiple fentanyl analogues (very short acting) that can be added into a base product of longer-acting poppy-based powder heroin. Although both varieties of opioids can be mixed with methamphetamine for a ‘speedy’ shot, China white is typically preferred for polysubstance use due to its superior solubility and ease of measurement and handling. Both can be smoked or injected, yet ‘goma negra’ is considered less wasteful to smoke because it releases psychoactive components efficiently when combusted.

## METHAMPHETAMINE

Unlike fentanyl or xylazine, Mexican methamphetamine entered the US market as a cheaper, autonomous product of purer and more potent quality than the stimulants formerly available in US inner-city street markets (e.g., cocaine, crack, and domestically ‘cooked’ methamphetamine). It did not enter camouflaged as did the fentanyl sold ‘as heroin’ east of the Mississippi River (Mars, Ondocsin & Ciccarone 2018). Mexican-sourced crystal methamphetamine is always sold as what it really is: ‘meth’ or ‘crystal meth.’ Most users we interviewed considered this new methamphetamine an unambiguously superior product than cocaine, with a lasting and satisfying high as well as a more potent initial ecstatic rush. It is therefore acting as a permanent replacement for cocaine among working class and street-based users for whom cocaine was always relatively expensive. Users today are not only switching from cocaine to methamphetamine. Many are developing stimulant habits that they never had before.

Mexican methamphetamine, consequently, is poised to revolutionize street drug markets in the eastern half of the United States in coming years (Ciccarone & Shoptaw 2022). The impact on public health is already evident, with psychostimulant overdose deaths in the United States rising by 625% in the US between 2012 and 2019 (Hedegaard, Minino & Warner 2020: 4). There is plenty of room for expansion east of the Mississippi River. The Centers for Disease Control and Prevention (CDC) report that ‘age-adjusted death rates for drug overdoses involving psychostimulants with abuse potential ranged from 1.2 [per 100,000] in the Northeast to 5.3 in the West (CDC 2019: 37).’ Mexican methamphetamine’s displacement of domestic methamphetamine is reflected in the precipitous decline in what the DEA calls ‘domestic methamphetamine laboratory incidents’ throughout the 2010s. In 2010, there were 15,256 domestic incidents, decreasing to only 890 by 2019 (DEA 2021: 22, Figure 14).

Recent public health studies show that co-use of methamphetamine and opioids is growing rapidly across the United States (Park et al. 2021; Ellis et al. 2018; Strickland et al. 2019). As we detail below, drug users insist that meth promotes risk-taking in sex, sex work, and other income-generating practices in addition to party/binge settings. It generates an ecstatic overconfidence in one’s ability to metabolize opioids safely. Co-use of methamphetamine and opioids has been associated quantitatively with higher prevalence of Hepatitis C (Shearer et al. 2020) and blood-borne virus infection (Cai et al. 2020). Rising rates of co-use of methamphetamine, heroin, and fentanyl in recent years are reversing a two-plus decade decrease in HIV incidence among injection drug users in the US (CDC HIV 2018). Other infectious diseases rising among injection drug users include soft tissue infections and endocarditis (Ciccarone 2021: 347).

Consistent with US patterns of segregation, these pathologies are unevenly distributed along the fault lines of race/ethnicity/indigeneity, gender, class, and sexual orientation (Bourgois et al. 2006). The highest overdose death rates involving meth in 2018 affected Native American men, at 26.4 deaths per 100,000 persons, followed by Native American women (15.6) and non-Hispanic white men (12.6) (Han et al. 2021a: 565). The spread of Mexican methamphetamine appears to be having an impact similar to the explosion of crack in the 1980s, sweeping new generations of drug users into chronic use of stimulants with a cheaper, more potent, readily available smokable and injectable substance. Meth appeals to an even wider range of demographics than did crack in its late-1980s and early-1990s heyday. Meth consumers include white and Native American street-based users and has long-since appealed to a range of cross-class/ethnic users of all ethnicities in queer/MSM scenes (Nerlander et al. 2018; Bluthenthal et al. 2017). Mexican meth in Philadelphia appears to be triggering a class- and ethnic-based split in the heterosexual stimulant market, with wealthier white users maintaining preference for cocaine, Latino and African American street-based users maintaining preferences for crack, and working class white users initiating or shifting to stimulant use through crystal meth. That said, Mexican methamphetamine’s presence in street drug markets has become so pervasive in the last half-decade that US-wide overdose death rates associated with psychostimulants are rising for most demographic groups, including non-Hispanic Black men (1.5 deaths per 100,000 in 2014 versus 6.4 per 100,000 in 2018), Hispanic men (2.6 in 2014 vs. 6.6. in 2018), and women (1.5 in 2014 vs. 4.5 in 2018) (Han et al. 2021a: 565).

## METHAMPHETAMINE IN PHILADELPHIA

Mexican methamphetamine took the Philadelphia open-air drug market by storm in 2020. A new meth user described the changes in May 2021:

‘Methamphetamine just hit Philadelphia real hard this year. That’s like taken off. You got a totally different ballgame because now you got people who have been up for like 4–5 days, 6, 7, 8 days and they’re losing their mind, you know. They’re like talking to shadow people and by Day 7 they’re like siding with them, plotting and scheming with them. At first you really think it’s funny but it’s not. There’s guys out here that look like ballerinas. People can’t control their bodies, their movements, or what they do. Irrational thoughts turn into actions. You can’t tell yourself “No, don’t do it.” If you think something, your body just, your mind just does it. You know what I mean? Usually when you think bad things you just think bad things, but when a person’s been on meth for a while, when they think bad things they start doing bad things whether it’s like stealing from a friend or whatever. A lot of people are waking up in jail and they don’t remember what they did.’

Mike, 28, White, opioid and methamphetamine injector

The relative newness of methamphetamine outside LGBTQ+ venues in Philadelphia manifests in its uneven availability in open-air markets. While most sales spots in North Philadelphia/Kensington offer ‘dope,’ only about a third also offer methamphetamine. Users—predominantly Whites—list several reasons for their new methamphetamine ‘habits.’ Meth helps to counteract fentanyl’s contraction of the opioid high, providing users the necessary time and energy to find money to buy more ‘dope’ before the onset of overwhelming withdrawal symptoms:

‘Fentanyl is so short-lived that we gotta get high every 6 hours. But sometimes, let’s say things were tough or whatever, if we were 10 hours in and you started getting sick, a nice shot of meth will hold you over for another 6 hours, as far as not being sick altogether. You just wouldn’t be as sick, I mean eventually it would turn against you but it would keep you feeling better to get up and make a move to make money.’

Suzy, 24, White, opioid and methamphetamine injector

Methamphetamine meshes well with street-based users’ income-generating strategies, primarily trading sex for money (for women) and scrapping metal (for men). These are predominantly nocturnal activities:

Mike: ‘Meth does its part too, because if you really don’t have the money, meth can keep you well for a little bit longer, it kind of will help you like, give you some time to gather up money to find some dope, you know what I mean? And you can stay up later, scheming to get money, or if you do your thing at night, boosting or whatever. I scrap, so a lot of my shit is nighttime stuff.’

This relationship between opioid/sedative withdrawal symptoms and methamphetamine remains significantly underexamined in the field of public health. In cities with different supply chains (such as Huntington, West Virginia, where FM conducted several days of ethnographic research in September 2019) users reported being able to minimize long-term opioid withdrawal symptoms by using methamphetamine. In Philadelphia, however, users insist that methamphetamine injection provides only limited respite:

Mike: ‘You’d be doubling down, basically. You’d be sending yourself to go into like The Hole, you know what I mean? Like you only get one extension [laughing]. That second one, you’re gonna be sketched in a hurry, you’re probably gonna be like… You’re gonna do something stupid or someone’s gonna help you eventually, like, it’s gonna go down.

The second shot of meth isn’t like the first shot, as far as helping you. Now it’s like going against you. Now you’re kinda super wired and you’re like high and sick now, you know what I mean? You’re still not feeling as bad as you would have being dopesick, but like it doesn’t really like… Put it this way, you’re not even gonna want to do more meth. It’s gonna go down. You’re gonna figure it out [find a way to get more “dope”].’

Fernando: ‘What if after those 6 hours you shot more meth?’

Stimulant preferences differ across ethnic boundaries and manifest as forms of ‘intimate apartheid’ (Bourgois & Schonberg 2009). The relatively few Puerto Rican users in the North Philadelphia street scene hang out near the predominantly African American encampments where crack continues to be the stimulant of choice and is thus more readily available. None of the three Puerto Rican users interviewed in the spring of 2021 knew what methamphetamine was. One Puerto Rican woman thought FM was referring to methadone when he asked her about ‘meth.’

## METHAMPHETAMINE IN TIJUANA

Methamphetamine is also used widely across Tijuana by numerous demographics, including regular consumers of street drugs and more casual recreational users. Cheap ‘*globos de cristal*’ [methamphetamine balloons] are available for as little as 25 pesos (about US$1). It is widely available in *conectas* [sales points] across the city. For example, in extreme proximity to the US pedestrian border crossing, White US monolingual, English-speaking crystal meth consumers can be found smoking for hours on dilapidated couches in graffiti covered rooms. Entry-level workers in large US-owned maquiladoras (factories on the border producing numerous goods for export to the United States) reported using methamphetamine to help cope with long working hours and low wages that require many to work two or three jobs to make ends meet. It is commonly consumed by the friends, romantic partners, and other peripheral members of the opioid scene in *Zona Norte* [open-air markets near US pedestrian border-crossing]. Many local residents smoke methamphetamine for years before ultimately shifting to opioid- methamphetamine co-use.

Among the most marginalized, homeless opioid-using groups, especially Mexican deportees, who were the focus of our fieldwork, it was increasingly common (2018–2021) to mix small amounts of crystal methamphetamine into nearly every injection of ‘China white.’ However, the ratio of methamphetamine to opioids was small, often 1 part methamphetamine to 5–10 parts opioids. A typical user, consequently, might go through many bags of China white in a single day, while their globo de cristal might last several days. Chipping in a small touch of *cristal* can also be an important, albeit low-cost, bargaining chip to negotiate a small taste from a more costly opioid shot prepared by others later on.

Many reasons are given for the recent, widespread tendency to inject ‘speedies’ (injections combining opioids and methamphetamine). Many users report improved euphoria and increased delay of opioid withdrawal symptoms. Others assert that methamphetamine prevents opioid overdose, due to its stimulating effects. Nevertheless, a contingent of mostly older and exclusively black tar heroin consumers avoid *cristal* claiming it ruins heroin’s soothing euphoria:

‘Nah man, I don’t touch that synthetic crap [methamphetamine]. I like my sleep. Plus, it ruins the high. Takes away all the relaxation and gives me anxiety. Look, I’ve been using heroin for over 30 years cuz I love the way it makes me feel. I know lots of people around here are using meth and switching to that fake China white stuff, but I don’t understand why you would want to ruin the best feeling in the world.’

James, 55, male, White US monolingual English speaker, living in Tijuana for 20 years, injects only black tar heroin

## FENTANYL

Fentanyl has had a long, albeit intermittent, career in the US opioid supply (Ayres, Starsiak & Sokolay 1981). Its irruption into US drug markets in 2013/2014 unleashed the so-called ‘third wave’ of the opioid overdose epidemic. The first prolonged wave was provoked in the late 1990s—late 2000s by doctors over-prescribing opioid medications. The second wave (late 2000s—2013) occurred after US Congress changed opioid prescription formulations thereby propelling opioid-dependent prescription pill users experiencing withdrawl symptoms to seek the cheaper, more easily injectable, and potent heroin available in street markets nationwide. The fentanyl era revived the punitive thrust of US drug policy against street-based users and retail sellers at a moment when the tide had been shifting in favor of public health treatment/prevention logics (Karandinos 2018). The US continues to incarcerate more people than any other nation, with nearly half of Federal prison inmates confined on drug charges (The Sentencing Project 2021).

Fentanyl has spread unevenly throughout the United States. It first proliferated on the East Coast (Zoorob 2019), where it served as an adulterant that could be easily mixed—undetectable to the naked eye—into the powder formulations of heroin that have long prevailed in that part of the country. In the West, fentanyl has struggled to find a footing beside the solid, black tar form of heroin that has monopolized markets west of the Mississippi since the early 1990s (Ciccarone & Bourgois 2003). Westward expansion only began occurring recently and at a smaller scale than in the eastern US, although 2020 statistics reveal rising public health ravages (Shover et al. 2020). Notably, in 2018, the DEA detected fentanyl in only three of the hundreds of wholesale Mexican black tar samples they seized (DEA 2020: 5). Because of these visible characteristics, fentanyl had to enter the West Coast as a bona fide standalone opioid instead of as an adulterant. It was explicitly advertised by sellers as fentanyl—rather than passing for ‘dope’ or ‘heroin’—in contrast to many eastern street markets (see also Ciccarone 2021: 346).

While fentanyl shipments from China were prominent in the initial stages of the ‘third wave,’ Mexican fentanyl production almost fully supplanted Chinese production in the mid-2010s (DEA 2021: 14–18). Most of the fentanyl entering the country appears to be crossing Southwestern border points with purity levels around 1.5–10% (DEA PFD 2020b: 8), reflecting the toxic potency of the substance. As noted earlier, fentanyl is rarely mixed with heroin at wholesale international or even regional distribution levels.

Fentanyl is a particularly versatile product because it can act as an enhancer, a counterfeit, and a substitute for any other opioid, including heroin and opioid prescription pills. That said, experienced users throughout the eastern half of the United States have belatedly developed the ability to detect the presence (not the concentration) of fentanyl in the sedative supply through taste, texture, and quality and length of fentanyl’s initial opioid rush and subsequent high (Mars, Rosenblum & Ciccarone 2018; Zibbell et al. 2021; Duhart Clarke, Kral & Zibbell 2022). There have been widely publicized incidents of fentanyl-laced stimulants such as cocaine and methamphetamine, but this kind of cross-category mixing in the retail supply has been uneven and may be caused by error—or one-off experimentation—in packaging mills (Park et al. 2021: 2). Data from a new drug testing program in Philadelphia show that cocaine samples in the city sometimes contain miniscule traces of fentanyl. Out of 24 samples of cocaine tested by the program between March and September 2022, only 2 contained trace levels of fentanyl. In contrast, ironically, crack and methamphetamine remain the least adulterated substances in the street supply (CFSRE 2022a; CFSRE 2022b; Whelan 2022), suggesting distinct wholesale/retail market supply chains.

## FENTANYL IN PHILADELPHIA

Following 30 years of stability in heroin’s street retail price ($10/packet), adulteration with synthetic sedatives, primarily fentanyls but also xylazine, suddenly transformed the retail pricing structure of ‘dope’ in North Philadelphia in the late 2010s. ‘Dope’ packets dropped from $10 to $5 at most salespoints by mid-2021. From 2007–2018, ‘bundles’ of heroin (a wholesale supply pre-packaged for individual street sales) generally consisted of 10–14 $10 packets sold to street-level wholesale customers for between $100– $140. In mid-2021, ‘bundles’ of dope contained 16 ‘bags’ in postage stamp-sized glassine envelopes and only cost $80, sometimes discounted to $65–75. Meanwhile, the volume of retail bags remained constant at 0.03 grams (Bourgois et al. 2018: 54) but the substance itself completely changed.

In 2021, a user gleefully described this 50% drop in the unit price:

‘One street, Wind Street, they put nicks out [“nickles” or $5 bags] and they kind of spread like wildfire. I was used to getting 6 for $50, I get out of jail and go back to getting high and I couldn’t believe it. Bags were $5, I felt I was in like dope paradise.’

Users’ celebration of ‘dope-paradise,’ however, is tempered by their recognition that the drop in prices also drives them to purchase more bags because they are now consuming opioids with a shorter metabolic half-life, requiring more frequent rounds of injection. Economically and pharmacologically, fentanyl in the mid-2010s—early 2020s evokes the sudden inexpensive access of crack in the mid- 1980s to early-1990s prompting profitable chronic consumption patterns (Bourgois 1996: 352, n.2). Fentanyl can be smoked but has entered injection markets most explosively. More frequent injections logistically exacerbate the negative public health consequences of fentanyl given the absence of safe injection sites, limited treatment access, and discretionary intermittent police harassment for paraphernalia possession—conditions that increase the re-use and sharing of dirty needles.

Triangulating municipal public health data and an investigative journalist’s year-long study suggest that the entirety of Philadelphia’s street-level ‘dope’ supply contains fentanyl (Johnson et al. 2021: 2; Moraff 2018; Whelan 2022; CFSRE 2022a; CFSRE 2022b). Increasingly, heroin is disappearing and fentanyl is the only consistently available opioid in street markets. One user carefully explained the “disappearance” of “real heroin” and its promotion of more expensive iterative use:

‘It’s $5 now and it’s the same amount, but you’re not paying for that quantity of dope; you’re paying for fentanyl, which is cheaper to make. It’s stronger. It hits you harder faster, but it’s over sooner, so you gotta cop again. Fentanyl’s every 6 hours you need a bag. Whereas like heroin you could probably do two shots a day and you can maintain, like 10 hours, 8 hours till you start to feel sick. We all know when we’re not high and we all know when we’re gonna start to get sick.’

## FENTANYL IN TIJUANA

The availability of both powder heroin and black tar in Tijuana rendered fentanyl adulteration much more variable than in Philadelphia. This local variability largely results from the existence of multiple groups vying for control of local drug supplies. Tijuana has long been a key transit point for smuggling wholesale narcotics into the US. Several splintered groups claiming affiliation to rival drug trafficking organizations struggle for control of both local sales and cross-border smuggling. They compete with both firearms and political influence. Our ethnographic data indicate that different suppliers add fentanyl to the drug supply in distinct concentrations and at distinct points along the distribution chains. This local landscape of extremely uncertain potency exacerbates overdose rates. In ethnographic conversations, drug users involved in low-level retail sales described adding fentanyl analogues at the local micro-neighborhood *conecta* level:

‘So we be getting these big bricks of like brownish powder right…and they real hard, and we gotta grind ‘em up in the food processor. And you know that brown shit stretches, it’s got some good legs even though it’s weaker. I mean, it don’t get me high no more, not by itself. So then we gotta mix in the fentanilo too, and it’s like kinda dusty and whiter. And that shit is bomb… cuz we can’t be selling that pure fentanilo no more, cuz waaaay too many fools be dying off that stuff.

And then you know there’ll usually be like different mixes of the brown and the fent, depending on where it’s going, you know, to the US or if it’s staying here. But it’ll depend on the motherfucker that cooks it up too, so that’s why all the sudden you’ll see so many fools dying out here, cuz the shit be good dawg!’

## XYLAZINE

Over a decade after the first reports of heroin’s frequent adulteration with xylazine in Puerto Rico (Rodriguez et al. 2008, Torruella 2011), this veterinary analgesic, sedative, and muscle relaxant designed for heavy animals penetrated Philadelphia’s opioid supply in the mid-2010s. According to Philadelphia’s Medical Examiner’s Office, xylazine was detected in less than 2% of cases of fatal heroin/fentanyl overdoses in 2010 and remained low until starting to rise in 2016 and 2017 (both 7.4%) and then accelerated from 14% in 2018 to 26% in 2020 and then saturating the “heroin” market by mid-2021 (Philadelphia Department of Public Health 2021; Johnson et al. 2021). A Puerto Rican public health study conducted between 2005 and 2007 documented xylazine adulteration on the island in cattle-ranching towns like Arecibo, Yauco, and Guanica (Rodriguez et al. 2008: 291). Significantly, on the US mainland, the first places to detect significant concentrations of xylazine in street opioid markets have been deindustrialized localities with large numbers of segregated Puerto Rican residents, including Philadelphia, Chicago, and cities and towns throughout Connecticut and Maryland (Friedman, Montero & Bourgois 2022).

Street-based opioid users in Philadelphia are well aware of xylazine’s growing presence in the local opioid supply. Prevention Point, the local harm reduction non-profit, was circulating flyers about xylazine adulteration throughout the spring and summer of 2021. Spanish-speaking Puerto Rican users call xylazine ‘anestesia de caballo [horse tranquilizer],’ while English speakers term it ‘tranq.’ According to users, xylazine adds to retail opioids some of the qualities lost when fentanyl’s increasingly displaced heroin:

‘Fentanyl is such a short-lived high, that the high… It’s a good high but it’s so short that the nod is over real quick and you get sicker faster. See, the tranq extends the high, it gives the dope more of a heroin effect, it’s a good rush with the heroin-like “legs” [duration of high].

But they straight put bags out there that are just all tranq. You shoot it, you feel no rush. Tranq-fent is like you shoot it; you get the rush of the fentanyl; then the tranquilizer comes in; you nod; and you fall asleep. A straight tranq bag is like, you shoot it; you get no rush; you’re sitting there for a second talking; and then you’re waking up 2–3 hours later in a weird position.

Like one case, I lit a Newport [menthol cigarette]; I shot a bag with a Newport; I woke up with a hole of Newport burnt into my stomach [showing the scar, laughing].

You could literally drown in a half-inch of water if you did a tranq bag and you fell out.’

Tranq it stretches fentanyl out. It’s sought after. Certain stamps [salespoints with ink-stamp logos on packets] that are known for tranq have better business. Some people don’t like it. I like a good fentanyl-tranq bag. You know a bag got tranq in it because you’ll shoot it and you’ll get dry mouth right away and you know, you taste it [shaking his head in disgust].’

Tom, male, white, 35-year-old opioid and meth injector

Users are also concerned about the new types of skin lesions affecting them since xylazine’s arrival on the scene. The Puerto Rican public health literature refers to these lesions as ‘ulcers,’ specifically distinguishing them from abscesses (Rodriguez et al. 2008; Reyes et al. 2012). One user in Philadelphia emphatically explained:

I was getting abscesses [ulcers] more from the dope than I do from the meth. Right now, everybody is getting these scabby sores all over their bodies… and many of them don’t shoot meth. So it’s from the dope. People say it’s krokodil [tranq]. I got some friends out here that got really torn up by it, you know they got holes in them, abscesses, basically it’s like the body is rotting. It’s big! You don’t really hear too much about it, people here are losing limbs like with gangrene.’

Suzy, 24, White, opioid and methamphetamine injector

Virtually all opioid users complained emphatically often at a loss for words to convey the severity of combined xylazine/fentanyl withdrawal symptoms:

‘You had the dope, then the fentanyl, now it’s the ‘tranq-fentyn.’ Tranq is like the tranquilizer, the rhinoceros tranquilizer, the horse tranquilizer. Cooked up and it’s broken down and it’s added to the dope and we seek that out. Our habits are fentanyl and tranquilizer. It’s a totally different withdrawal, you know—a 30 mg methadone taper for 5 days doesn’t do it, they have to put you on methadone on a high dose real quick to even just stop… The tranquilizer is just a different game—your head chimes kind of like wind chimes.

It’s a totally different withdrawal, you think you’re over it and it comes back, it has like a second wave to it I wanna say, like you know, the anxiety, I don’t know, it’s hard.

Heroin is like going seven days and seven nights against Tyson, you know, like humble. Tranq-fentyn is more like let’s say like 14 days getting pummeled by 20 little kids. It’s annoying, the headaches… you don’t even get up to throw up, or to get a shower. It puts you down. The times I’ve been arrested, I’ve withdrawn in jail from methadone, suboxone, heroin, fentanyl… Fentanyl… it’s the tranq too, I mean, you get so dehydrated that your body, your muscles don’t even work, so you can’t even move and the trance, the migraines, and then you have the actual ‘hallusions,’ they’re like dreams but they’re not. You’re not sleeping for 30 days, you know? You’re going through it. You get delirious, you do.’

Alex, male, white, 33 years old, opioid and meth injector

In short, xylazine adulteration addresses several of the disadvantages that fentanyl enhancement/replacement of heroin introduced for opioid consumers and suppliers. As a low-cost opioid, fentanyl is a relatively effective adulterant and even substitute for heroin, but it poses two problems: it has a noticeably shorter effect, and it cannot act as a diluent or as the bulk of the substance sold to consumers because it is too potent a respiratory depressant e.g., inadvertently killing more customers. Xylazine, on the other hand, is both a long-acting sedative and a diluent. It is legal and cheaper than fentanyl. It extends fentanyl’s sedative effect, but its impact on breathing is weaker than that of synthetic opioids. This allows local wholesalers in suburban packaging houses to combine larger quantities of xylazine with minuscule amounts of fentanyl, satisfying the demand for an opioid agonist and an initial ecstatic rush of opioids, and enabling a longer sedative high. Increasing the concentration of xylazine enables reducing the quantity of fentanyl mitigating risk of fatal overdoses that shrink the customer base and attract police attention. As a result, by mid-2021, Philadelphia’s retail “dope” market offered sedatives consisting on average of 30–40% xylazine and 2–10% fentanyl with virtually no heroin (Whalen 2022).

Xylazine obviously presents unique severe problems, however. These include new varieties of skin lesions, risk of violent victimization and bed sores (or more likely concrete bruises) while heavily sedated, and according to all users interviewed in the spring of 2021, more severe withdrawal symptoms. Even though its respiratory effects are weaker than fentanyl’s, xylazine *is* also a respiratory depressant. Ethnographic and medical reports (Ehrman-Dupre et al. 2022) reveal that sedative users in Philadelphia have developed a combined dependence on fentanyl (an opioid) and xylazine (an alpha-2 adrenergic agonist that does not respond to naloxone/Narcan overdose reversal). Widespread physical dependence on these two substances will require public health practitioners to proactively reformulate their best practices for the treatment of overdose, withdrawal, soft tissue infections and violence prevention. It necessitates an expansion of the currently available arsenal of testing technologies that might allow users to detect the presence and concentration of fentanyl *and* xylazine in their drug supply (Reed et al. 2022).

## CONCLUSION

In examining the recent, far-reaching changes to narcotics markets from the mid-2010s to mid-2021, in two very different cities on opposite sides of the North American subcontinent, this article seeks to shed light on underexamined features of the US ‘war on drugs.’ It argues for expanding the purview of social science and public health research beyond individual drug use behaviors to include other key actors and aspects of the wholesale-to-retail market supply chains. Social science researchers and public health practitioners are only just beginning to develop robust understandings of hemispheric, national, regional, local, and neighborhood-level supply chains. Translationally, they could leverage this knowledge to cultivate diverse applied strategies of non-punitive engagement with criminalized actors across narcotics supply chains to decrease harms. Public health and social scientists could innovatively transcend taboo “rule of law” hostilities against criminalized drug smugglers/sellers that bias most state officials, scholars, medical practitioners, service providers, and “human subjects” Internal Review Boards. We need to collect/analyze valuable complex upstream drug supply chain data to benefit vulnerable street-based drug consumers. By cultivating relationships with criminalized actors, a social science-informed public health could develop innovative upstream harm reduction interventions capable of regulating narcotics supplies and wresting resources and jurisdictional capacities away from the often incompetent, delayed, and ultimately punitive logics of law enforcement that promoted the inexpensive public availability of highly toxic synthetic street drugs across much of North America.
